# Adapting a mental health program for people with multiple myeloma and smouldering myeloma: a consumer-informed feasibility study

**DOI:** 10.1007/s00520-026-11163-2

**Published:** 2026-09-08

**Authors:** Natalie Tuckey, Matthew Iasiello, Melissa Cantley, Joep van Agteren, Hayley Beer, Daniel B. Fassnacht, Imogen Ramsey, Laura C. Edney, Rayan Saleh Moussa, Luke Grundy, Mark Ryan, Kathina Ali, Hannah R. Wardill

**Affiliations:** 1 Supportive Oncology Research Group, Precision Cancer Medicine Theme, South Australian Health and Medical Research Institute, Adelaide, South Australia Australia; 2 School of Pharmacy and Biomedical Science, College of Health, Adelaide University, Adelaide, South Australia Australia; 3 Be Well Co, Sydney, New South Wales Australia; 4 Precision Cancer Medicine Theme, South Australian Health and Medical Research Institute, Adelaide, South Australia Australia; 5 Myeloma Research Laboratory, School of Pharmacy and Biomedical Science, College of Health, Adelaide University, Adelaide, South Australia Australia; 6 School of Public Health, College of Health, Adelaide University, Adelaide, South Australia Australia; 7 Myeloma Australia, Parramatta, New South Wales Australia; 8 School of Health, University of the Sunshine Coast, Sippy Downs, Queensland Australia; 9 Caring Future Institute, Flinders University, Adelaide, South Australia Australia; 10 Flinders Health and Medical Research Institute, Flinders University, Adelaide, South Australia Australia; 11 Centre for Improving Palliative, Aged and Chronic Care Through Clinical Research and Translation (IMPACCT), Faculty of Health, University of Technology Sydney, Sydney, New South Wales Australia; 12 NeuroUrology Research Group, Flinders Health and Medical Research Institute, College of Medicine and Public Health, Flinders University, Adelaide, Australia; 13 Consumer Advocate, Byron Bay, New South Wales Australia

**Keywords:** Multiple myeloma, Smouldering multiple myeloma, Mental health, Mental wellbeing, Consumer-informed

## Abstract

Consumer involvement in mental health interventions is recognised as essential to producing research that is relevant, acceptable, and ultimately more effective. Here, we report on a mixed-method pilot study to evaluate the suitability of an existing mental health program for people living with incurable blood cancer, multiple myeloma, and precursor form, smouldering multiple myeloma. The study sought to identify consumer-informed recommendations to guide the future adaptation of a bespoke mental health program for this population who face significant psychosocial distress due to disease and treatment-related symptoms and burden. Twenty consumers (38–72 years) with multiple myeloma (*n* = 16) and smouldering multiple myeloma (*n* = 4), as well as Myeloma Australia nurses (*n* = 4), pilot-tested an existing online mental health program—the *Be Well Plan*. After program delivery, feedback surveys and semi-structured focus groups were used to inform future program adaptation. Pre-post assessment of empowerment, mental wellbeing, anxiety and depression were analysed to identify preliminary signals of change and inform future trial planning. Satisfaction with and feasibility of the program was encouraging. Qualitative thematic analysis of post-program feedback revealed several themes including the importance of connecting with other consumers, the need for myeloma-specific adaptation, and delivery, facilitation, and clinical implementation considerations. Univariate ANOVAs indicated significant improvements with large effects in both mental wellbeing (*η*_p_^2^ = .24) and empowerment (*η*_p_^2^ = .25), with non-significant effects on anxiety and depressive symptoms, providing important insights for future adequately powered trials. This study provides preliminary evidence of the feasibility and acceptability of a mental health program which will guide the adaptation and testing of a future myeloma-specific program, ensuring it is person-centred and designed for optimal sustainability, implementation and impact.

**Supplementary Information:**

The online version contains supplementary material available at https://doi.org/10.1007/s00520-026-11163-2.

## Introduction

Multiple myeloma (MM) is an incurable, relapsing–remitting blood cancer affecting plasma cells. Smouldering multiple myeloma (SMM) is an asymptomatic precursor stage with patients closely monitored through a “watch and wait” approach to identify signs of progression to MM. People living with MM often experience significant vulnerability, not only due to their compromised immune systems and treatment side effects but also due to the overwhelming psychological distress associated with living with an incurable illness [1]. The substantial symptom burden, characterised by chronic pain, fatigue, and other physical symptoms, contributes to a reduced health-related quality of life (HRQOL) [2]. The psychological impact for those with MM is considerable, arising from the need to adapt to the progressive disease trajectory, ongoing treatment demands, physical impairments, and prognostic uncertainty, with approximately half of newly diagnosed patients reporting clinically significant symptoms of anxiety and/or depression [3]. Similarly, those diagnosed with SMM also face heightened psychological distress as a result of the perceived risk of progression to active MM and the emotional burden of having to wait for the disease to progress before being able to commence treatment [4]. As such, psychological challenges appear at various stages of the disease trajectory, including initial diagnosis, treatment, relapse monitoring, and end-of-life care [5]. These experiences highlight the urgent need for accessible and effective psychological support tailored to the myeloma community [6, 7].

Increasing evidence demonstrates that mental health interventions in cancer populations can significantly enhance coping skills and psychological resilience in adapting to disease [8, 9] as well as the management of physical symptoms [10–12]. For people living with a MM diagnosis, whose disease management often includes ongoing cycles of highly toxic therapy, frequent monitoring and sustained uncertainty, mental health interventions have shown to improve quality of life [13]. While it is encouraging to see a growing number of psychological interventions for people living with MM, few studies have been developed with an explicit focus to address mental wellbeing, and few have deliberately engaged with the myeloma community to co-design these interventions [13–15]. This is a missed opportunity, as meaningful consumer involvement to improve the way new interventions are designed and delivered ensures relevance and optimisation to address consumer needs [16]. This study also has relevance to implementation science, particularly in relation to identifying factors that may influence future implementation and sustainable scale-up [17].

Another key aspect in successful research translation is a rigorous pre-implementation evaluation to guide the successful intervention uptake and sustainability [18, 19]. These evaluations not only provide valuable insight into a potential anticipated effect—vital for efficient trial design, e.g. power calculations—but they also provide practical insights that can inform iterative refinements beyond those that can be attained from a priori patient-led co-design, thereby increasing the likelihood that programs will be effectively integrated and sustained within the desired population. Understanding these dimensions prior to more rigorous testing is particularly important for complex populations like those living with MM, where the diverse, physical and psychological challenges experienced require adaptable and individualised support solutions.

The aforementioned gaps in mental health research and initiatives for people with MM, identified in patient forums organised by the peak Australian advocacy organisation National body, Myeloma Australia, underscore the critical need for psychological support tailored to those living with MM. Our team has previously tested an existing, evidence-based mental health intervention—the *Be Well Plan* (*BWP*), which was designed to be used in the general population—in breast cancer survivors, showing clear benefits in improving mental health outcomes [12]. The current study builds on to this work in pilot testing [20] whether the *BWP* is beneficial for people living with MM and SMM and what adaptations would make it suitable for this cohort. Conducted in partnership with MM consumer advocates and Myeloma Australia, it assesses the feasibility and acceptability, and gathered in-depth participant feedback to inform a future myeloma-specific mental health and wellbeing program.

## Materials and methods

### Study design

The study used a mixed methods design, combining quantitative pre-post outcome measures with qualitative evaluation survey feedback, individual interviews and focus groups. The research aims were addressed using both quantitative and qualitative measures, each of which was analysed independently before they were integrated, to look for convergence (e.g. how pre-post data aligned with consumer feedback); divergence (e.g. how the pre-post data may not have aligned with consumer reflections on their mental health); and expansion (e.g. using qualitative feedback to expand the consumers’ experiences) [21].

The study was guided by the Australian National Health and Medical Research Council (NHMRC) Framework for effective consumer and community involvement in research and the South Australian Medical Health and Research Institute (SAHMRI) Framework for consumer and community engagement (SAHMRI and Health Consumers Alliance of South Australia). A consultation process was undertaken prior to the commencement of the intervention with clinicians, consumers, and researchers to determine whether the *BWP* including study design, materials, and training content were appropriate for people diagnosed with MM and SMM. Meetings were held with consumer advocates (D.S and M.R), nurses from Myeloma Australia, Health Translation SA Consumer Engagement Group, experts in culturally and linguistically diverse (CALD) populations and experts in mental health and wellbeing programs design. Stakeholders provided critical insight into the recruitment approach and inclusion criteria for the study and agreed that the *BWP* was likely to be suitable for this population. As the intervention has been successfully implemented in a wide variety of contexts [22], including a cancer setting [12], and considering it focuses on the development of a personalised wellbeing plan, it was decided that a prior adaptation of the intervention was not necessary before commencing this study and was intentionally deferred until after the feasibility testing.

Ethical approval for this study was obtained from The Adelaide University Human Research Ethics Committee (H-2024-205) in accordance with the principles outlined in the Declaration of Helsinki.

### Consumers

Eligible consumer participants were adults (aged 18 years or older) with a self-reported diagnosis of MM or SMM at any stage, who were fluent in English, and residing in Australia. No further exclusion criteria were applied. The study was advertised to potential consumers through the Myeloma Australia monthly newsletter and targeted emails to members. To inform the next phase of co-design and facilitation of the myeloma-specific program, nurses from Myeloma Australia were also invited, based on capacity and interest, to participate in the online program.

### Intervention: Be Well Plan (*BWP*)

A comprehensive overview of the *BWP* and its theoretical foundations can be found in van Agteren et al. [23]. The *BWP* is a group-based, evidence-based mental health and wellbeing program that is typically live-facilitated over 5 weekly sessions. The intervention has previously been studied by Fassnacht et al. [22] and tested in a pilot study by Tuckey et al. [12] among women diagnosed with Stages I–IV breast cancer. Sessions follow a structured format incorporating psychoeducation, guided self-reflection, and peer-to-peer sharing, with delivery either online or face-to-face. During the program, consumers develop an individualised mental wellbeing plan by learning about and selecting from 30 evidence-based mental health and wellbeing activities, each stemming from the insights of a systematic review [24] showing the positive impact of different evidence-based therapeutic approaches on improving wellbeing, including Cognitive Behavioural Therapy (CBT), Acceptance and Commitment Therapy (ACT), mindfulness-based practices, and positive psychology [24]. Consumers also complete the *Be Well Tracker* [22], a brief survey providing personalised feedback on mental wellbeing, resilience, strengths and distress aimed to develop insight and self-reflection [24] at pre-intervention and before session 4.

### Procedure

#### Expression of interest and consent

Consumers completed an online expression of interest including general demographic questions (age, gender, location, ethnic group, time since being diagnosed with MM or SMM) and provided written informed consent to take part in the study (*N* = 37).

#### Pre-program preparation and baseline assessment

Consumers were contacted by phone to inform them of the details of the program and to ascertain their motivation to participate in the study to inform future recruitment strategies. Two weeks prior to the commencement of the program, they received a welcome email, a hard copy workbook and an invitation to attend an optional introduction session to meet the facilitator and troubleshoot any technology challenges. Consumers were also sent the link to complete outcome measures (described below).

#### Program delivery

The *BWP* was delivered online over 5 weeks via Zoom [25] by a clinical psychologist and certified trained *BWP* facilitator (K.A.) according to the standardised intervention protocol [23]. Myeloma Australia nurses (*n* = 4) were grouped into separate breakout rooms on Zoom during peer-to-peer group discussions, to enable consumers to share their reflections without influence. Although fidelity was not formally assessed, it was supported by the use of an experienced facilitator who delivered the intervention according to the standardised protocol.

#### Post-program assessments

Following the completion of the *BWP*, consumers were asked to complete the post-intervention outcome measures and an evaluation feedback survey (see Supplementary Methods).

#### Focus groups and interviews

Consumers were invited to attend a 90-min focus group within 2 weeks of program completion, with the option of an individual interview if they were unable to attend. The Myeloma Australia nurses and the *BWP* facilitator (K.A.) were also invited to be interviewed following the program completion. All focus groups and interviews were conducted via Zoom, audio- and video-recorded with consent, and facilitated by two trained facilitators (an experienced postdoctoral researcher and a Health Psychology Registrar) with experience in conducting focus groups (see Supplementary Methods for interview questions). Consumers were compensated with a $120 gift card upon completion of the focus group sessions, in accordance with institutional guidelines [26].

#### Program evaluation and feedback survey

At the conclusion of the program, consumers were invited to complete a brief evaluation and feedback survey to capture their experiences of the *BWP (*see Supplementary Methods).

### Outcome measures

Feasibility was evaluated using expressions of interest, time to recruit, baseline questionnaire completion, program commencement, attendance, completion of post-intervention outcome measures, qualitative participation, and reasons for withdrawal. Acceptability was evaluated using post-program satisfaction surveys, qualitative interviews, and focus groups, to assess perceived relevance, usefulness and implementation risks.

#### Mental wellbeing

Mental wellbeing was assessed using the 14-item Warwick-Edinburgh Mental Wellbeing Scale (WEMWBS) [27]. This scale measures both eudaimonic and hedonic aspects of mental wellbeing. Consumers respond on a 6-point Likert scale (0 = none of the time to 5 = all of the time), indicating how often they had experienced various thoughts and feelings over the past two weeks (e.g. “I’ve been feeling optimistic about the future”). Total scores range from 0 to 70, with higher scores reflecting greater mental wellbeing. Reliability estimates indicated acceptable internal consistency (*α* = 0.93).

#### Anxiety

Anxiety was measured using the 7-item Generalized Anxiety Disorder scale (GAD-7) [28]. Consumers rated how often they had experienced symptoms of anxiety over the past 2 weeks (e.g. “Not being able to stop or control worrying”) on a 4-point Likert scale ranging from 0 (not at all) to 3 (nearly every day). Scores of 0–4 indicate minimal anxiety, 5–9 mild anxiety, 10–14 moderate anxiety, and 15–21 severe anxiety, with a score of 10 or higher commonly used to indicate clinically significant anxiety symptoms. Total scores range from 0 to 21, with higher scores indicating greater anxiety severity. Reliability estimates indicated acceptable internal consistency (*α* = 0.87).

#### Depressive symptoms

Depressive symptoms were measured using the 9-item Patient Health Questionnaire (PHQ-9) [29]. Consumers indicated how often they had experienced symptoms of depression over the past two weeks (e.g. “Feeling negative about yourself or that you are a failure or have let yourself or your family down”) using a 4-point Likert scale ranging from 0 (not at all) to 3 (nearly every day). Total scores range from 0 to 27, with higher scores reflecting greater severity of depressive symptoms. Scores of 0–4 indicate minimal depression, 5–9 mild depression, 10–14 moderate depression, 15–19 moderately severe depression, and 20–27 severe depression, with a score of 10 or higher commonly used to indicate clinically significant depressive symptoms. Reliability estimates indicated acceptable internal consistency (*α* = 0.82).

#### Empowerment

Empowerment is an important element of mental wellbeing promotion, yet rarely measured as an outcome of interventions [30]. Empowerment was measured using a modified version of the health empowerment scale (HES). The HES itself is a modification from the original Diabetes Empowerment Scale, which was designed to capture psychosocial self-efficacy in the context of diabetes [31]. To modify, the word “health” was replaced by the word “wellbeing”, with resulting items such as “I am able to turn my wellbeing goals into a workable plan” and “I can try out different ways of overcoming barriers to my wellbeing goals”. A total score is calculated by averaging item responses. Higher scores related to greater empowerment. Reliability estimates indicated acceptable internal consistency (*α* = 0.89).

### Data analysis

Quantitative data from the post feedback survey and the pre-post outcome measures were analysed using IBM SPSS Statistics for Windows, version 29.0 (IBM Corp., Armonk, NY, USA). A repeated measures multivariate analysis of variance (MANOVA) was conducted to assess changes in the outcome measures from pre- to post-intervention, followed by univariate ANOVAs. Effect sizes were measured using partial eta squared (*η*_p_^2^), indicating the practical significance of the findings. *P*-values < 0.05 were considered significant, with cut offs for effect size being small *η*_p_^2^ = 0.01, medium *η*_p_^2^ = 0.06, and large *η*_p_^2^ = 0.14 [32]. A power analysis for a repeated measures MANOVA (within factors) indicated that the minimum sample size to yield a statistical power of at least 0.95 with an alpha of 0.05, a large effect size (*f* = 0.4), comparing two measures, with correlation among measures of 0.6, is 19. The primary analyses retained all participants who completed the pre-post-intervention measures. Post hoc exploratory analysis was subsequently conducted to examine the influence of comorbid health conditions occurring during the intervention period to consider the impact of significant health events on observed health outcomes.

Inductive thematic analysis as described by Braun and Clarke [33] was utilised to explore consumer feedback and acceptability of the *BWP*. Data included post-survey free-text responses, consumer, facilitator and nurse focus groups. All data transcripts were managed and analysed using NVivo (Version 15.0) [34] and analysed using the same inductive coding approach, including developing familiarity with data, generating initial codes, searching for themes, reviewing themes, defining and naming themes, and producing a report of findings.

Focus groups and interviews were audio recorded and transcribed verbatim. The transcripts were read by the first author (N.T.) and then re-read for familiarisation. They were initially read with no specific focus, though interesting features were noted. Codes were sorted into meaningful themes by two authors (N.T., M.I.). Themes and subthemes were then mapped, revised, and refined to ensure a good fit with the data.

To support integration of the mixed methods data, the first author then reviewed themes across all qualitative datasets to integrate the data into the themes to search for overlapping themes and collapsed these into the fewest themes possible and renamed them accordingly [21]. The final stages of analysis involved authors N.T., M.I., and J.v A. reviewing themes and subthemes to ensure that coded extracts were valid, logical, and reasoned. Reflective discussions were undertaken throughout the analytic process to consider researchers’ perspectives, given their involvement in the development of the intervention. Credibility of the analysis was enhanced through investigator triangulation involving multiple researchers during coding and theme refinement. The resulting themes were subsequently reviewed with consumers post-intervention to ensure they resonated with the participants’ experiences and accurately reflected the acceptability and relevance of the intervention.

## Results

### Feasibility

**Fig. 1:**
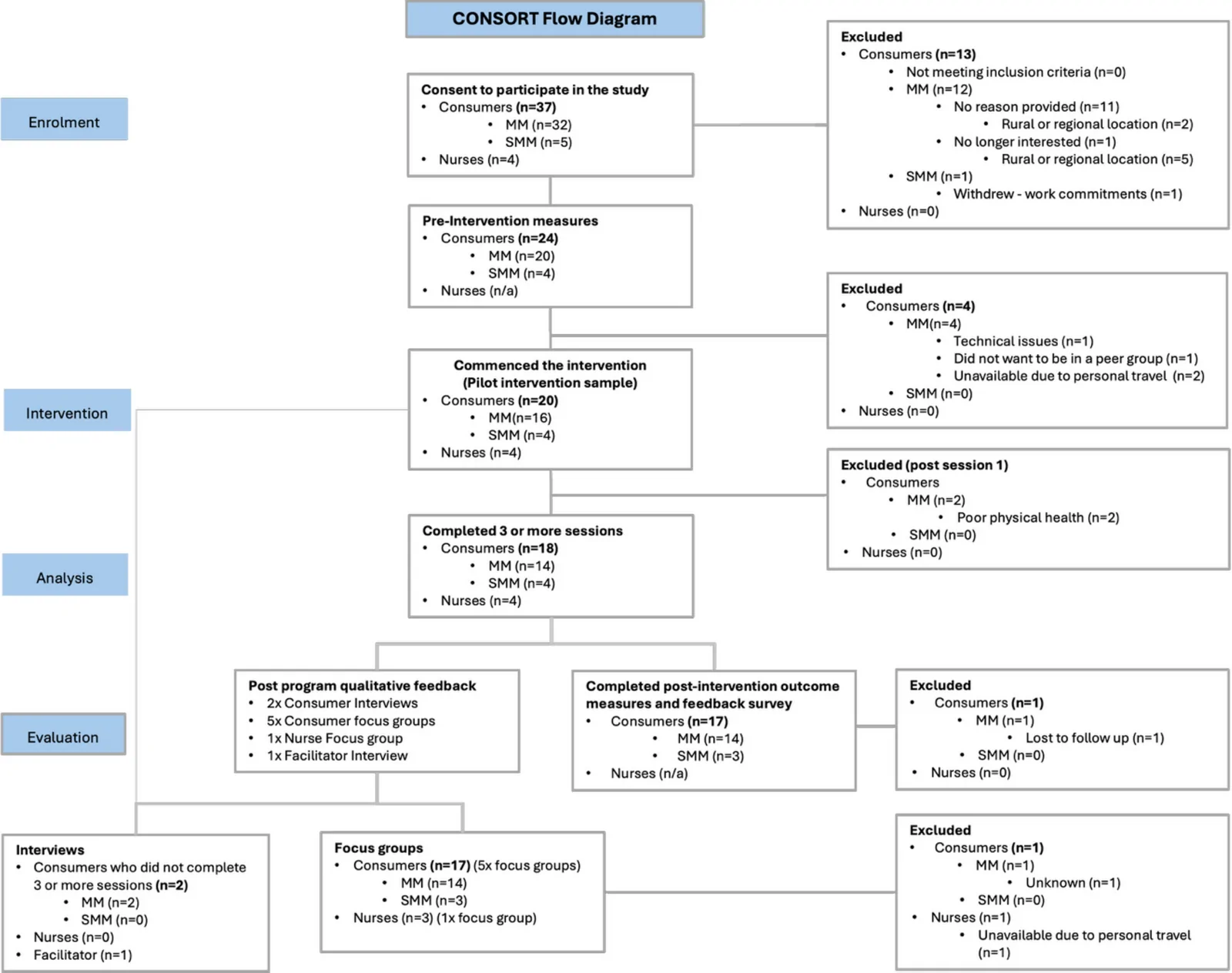
Consort flow diagram of the participation of consumers, Myeloma Australia nurses and facilitator

Over the 4-week recruitment period, of the 37 consumers who expressed an interest in the study, 24 (64.86%) completed the baseline questionnaire (Fig. 1); seven of the 13 who withdrew were based in a rural or regional location. Of these 24 consumers, 20 attended at least one session, with participants attending an average of 4.2 (out of 5) planned sessions and are considered the pilot intervention sample. All five intervention sessions were delivered as planned and per the intervention protocol. Four consumers who completed baseline assessments did not commence the intervention due to travel (n = 2), technical issues (n = 1), or not wanting to be in a peer group (n = 1). Four Myeloma Australia nurses also participated in the program and attended all five sessions.

A total of 18 consumers attended three or more sessions of the BWP. Two MM consumers withdrew prior to completing three sessions due to poor health. Seventeen consumers completed both the pre- and post-intervention outcome measures and therefore comprised the quantitative complete-case outcome sample. Those 17 consumers also completed the post-intervention feedback survey which included free-text responses and therefore comprised the quantitative complete-case outcome sample. Nineteen consumers contributed to post-program qualitative feedback: the two consumers who had withdrawn from the program due to health reasons took part in individual interviews, and the remaining 17 consumers took part across 5 focus groups. Motivations for participating in the study included a desire to contribute or give back to the myeloma community, a general interest in research, and an interest in growth and learning as an outcome of participation. All consumers identified as Caucasian/European. Consumer characteristics are presented in Table 1.

**Table 1:** Consumer characteristics from the pilot intervention sample (*n* = 20)

Characteristics	MM (*n* = 16)	SMM (*n* = 4)	Consumers (total) (*n* = 20)
Gender			
Male (*n*)	7	3	10
Female (*n*)	9	1	10
Age, mean (SD)	60.75 (10.9)	56.5 (2.9)	59.9 (9.9)
Location			
Metropolitan	12	3	15
Rural or regional	4	1	5
State/territory			
NSW	4	3	7
QLD	1	1	2
SA	6	0	6
WA	3	0	3
NT	0	0	0
VIC	1	0	1
ACT	1	0	1
Diagnosis date			
< 12 months	0	0	0
1–2yrs	3	2	5
2–3yrs	4	0	4
3–4yrs	4	2	6
4–5yrs	2	0	2
> 5yrs	3	0	3

### Acceptability from quantitative feedback survey

**Fig. 2:**
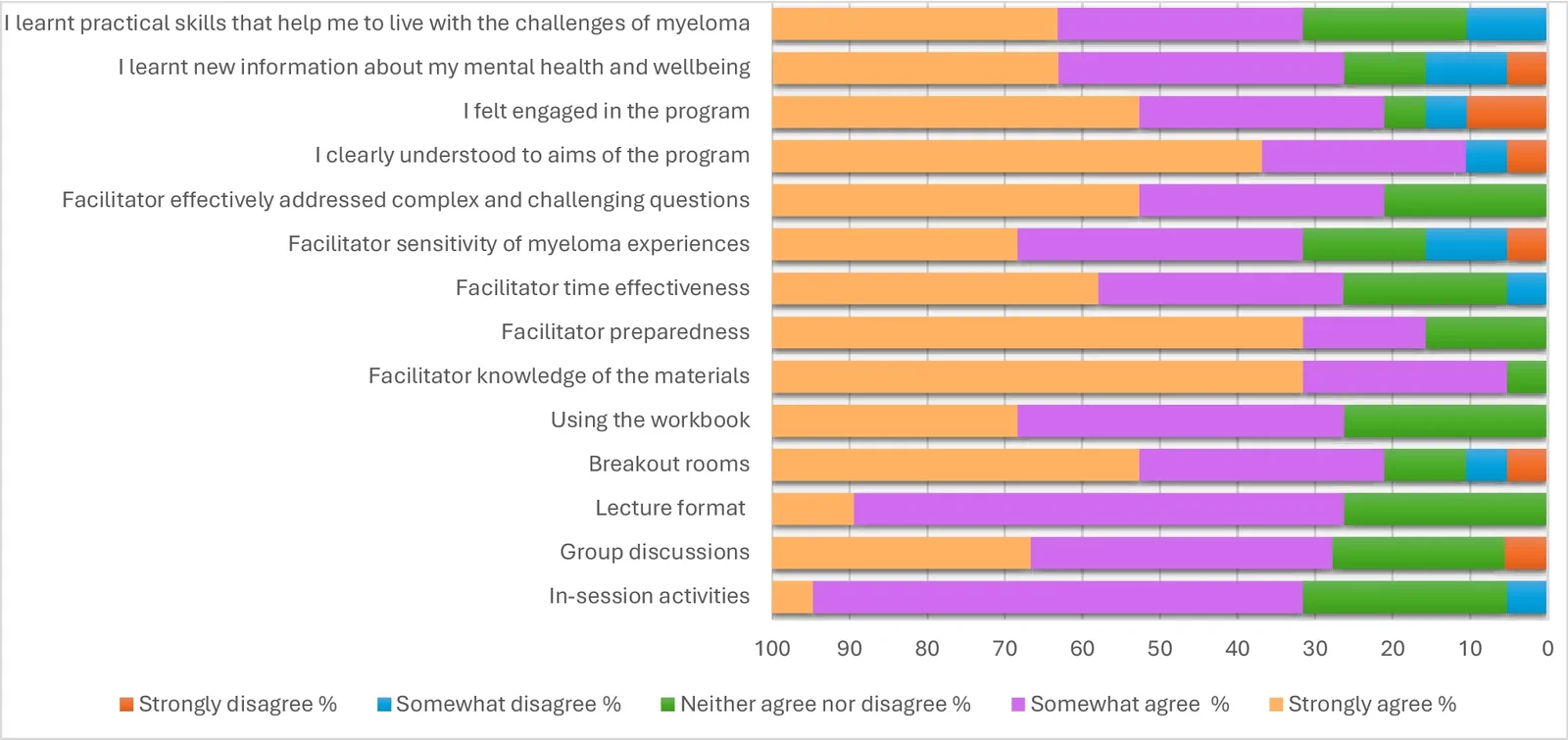
Post-program quantitative feedback from consumers on the program aims, relevance of content, and overall satisfaction (*n* = 17)

**Fig. 3:**
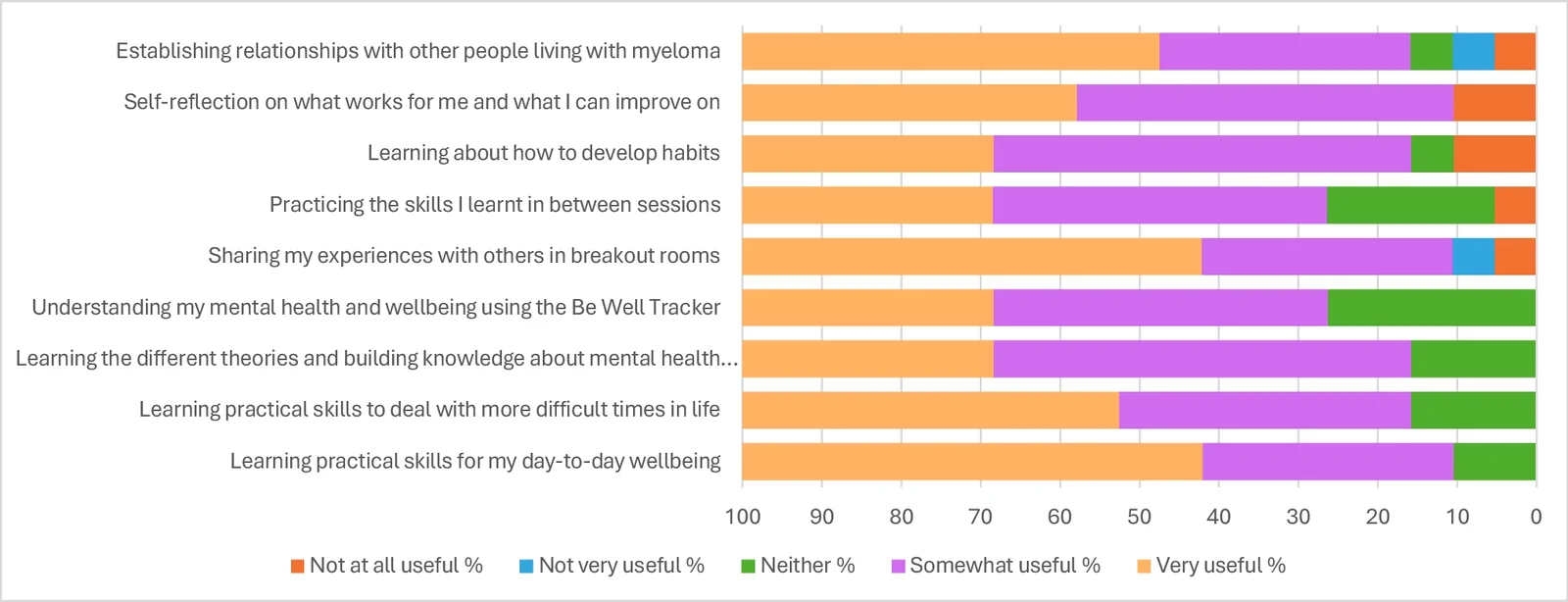
Post-program quantitative feedback from consumers on the usefulness of strategies (*n* = 19)

A total of 17 responses to the quantitative feedback survey were received (see Figs. 2, 3 and Supplementary Results). When asked, “Overall, how satisfied were you with the program?”, 79% of consumers reported being either *very satisfied* or *satisfied*. Additionally, 73.7% indicated that the program content presented new information to them most of the time, while 52.2% reported that it served as a useful reminder of existing knowledge. A total of 36.9% of respondents indicated that the program was not relevant to them some or most of the time. (See Supplementary Tables S1–S2). In addition to the survey, consumers contacted the program coordinator to report positive reflections about the program and suggestions for improvement.

### Acceptability from qualitative feedback

**Fig. 4:**
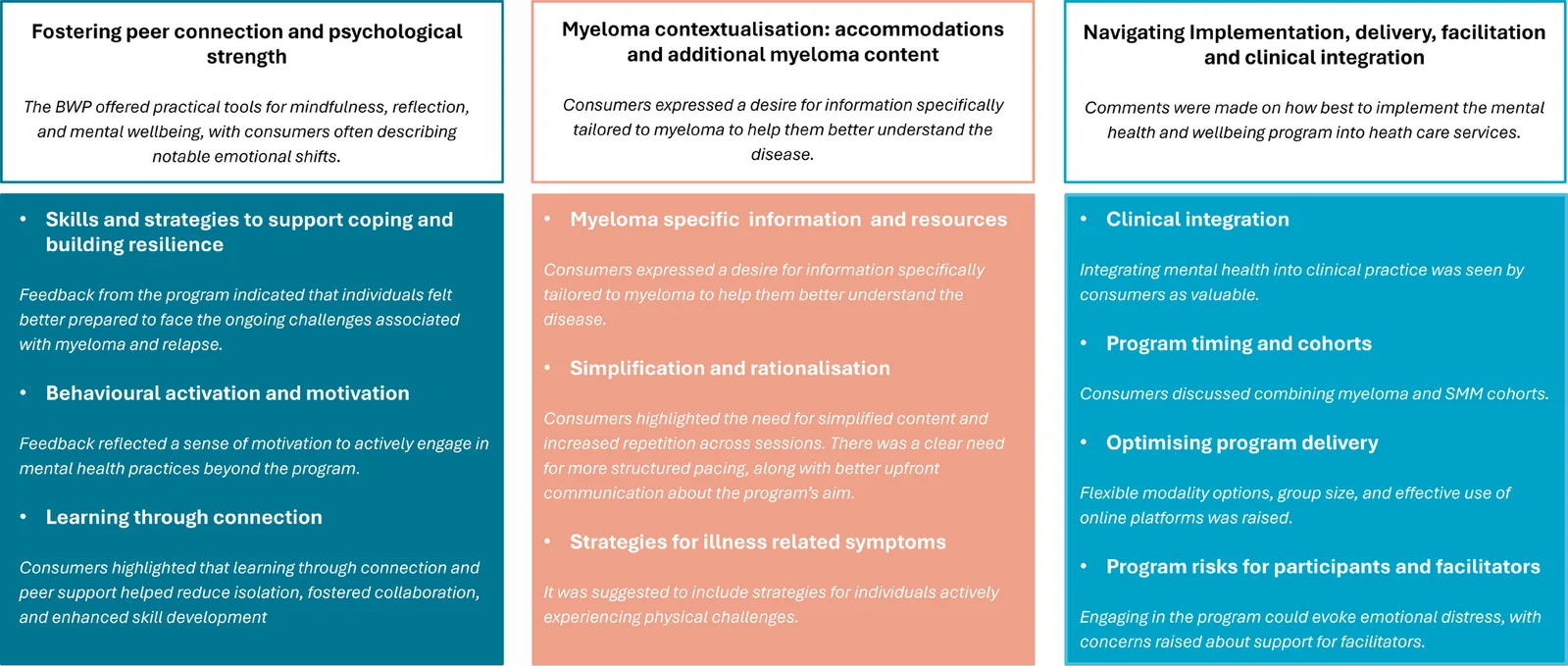
Qualitative themes, and sub-themes from post-program focus groups and interviews with consumers, Myeloma Australia nurses and the facilitator

Qualitative feedback was provided by consumers in post-program survey free-text responses in five focus groups, (*n *= 17), and two individual interviews with those consumers who did not complete the program. Additionally, one focus group with Myeloma Australia nurses (*n* = 3) and an individual interview with the group facilitator (*n* = 1) informed this analysis. Analysis of integrated qualitative data identified several opportunities to improve the program’s relevance and accessibility for people with MM and SMM, which are presented in these three themes: *fostering peer connection and psychological strength*; *adapting for relevance: integrating myeloma-specific insights*; and *navigating implementation* risks*: delivery, facilitation, and clinical integration* (Fig. 4). Sub-themes and verbatim illustrative quotes are presented in Table 2 (see Supplementary Table S4 for illustrative quotes of codes).

**Table 2:** Qualitative themes and illustrative quotes from post-program focus groups and interviews with consumers, Myeloma Australia nurses and the facilitator

Theme	Sub-theme	Illustrative quote
**Fostering peer connection and psychological strength** Consumers shared improved confidence in managing their mental health through peer support and learning practical wellbeing skills	Skills and strategies to support coping and building resilience	“This issue of looking after ourselves mentally as well as physically is so important during this wild journey of having myeloma and being treated for it. I believed if I was strong and fit mentally as well as physically, I would get through this, I treated it as a project during 2023–24. I know I have the tools now to use if I am faced with the recurrence of this cancer. I wish I had these tools and activities available to me when I was diagnosed and didn’t feel sick because the disease was asymptomatic”. (*Evaluation survey*)
	Behavioural activation and motivation	“I am now able to put in place activities that help my mental health. I practice meditation and find the 5,4,3,2,1 approach best. I like to do this outside. Each day I reflect on 3 things I am grateful for. I, also with my husband each evening, discuss our day and emphasise the positives of our day”. (*Evaluation survey*)
	Learning through connection	“It definitely gives you tools to manage when we get those times and working through those as a group. And these 2 guys, I was in their group with most of the time was great because we really got to know each other and we got to tease out stuff together”. (*Focus group 1*)
**Myeloma contextualisation: accommodations and additional myeloma content** This theme focuses on myeloma contextualisation, emphasising the importance of including myeloma-specific resources, strategies to manage physical symptoms, and simplifying content to make it more accessible	Myeloma specific information and resources	“I think it would be really helpful and also validating for the people in the sessions to have some explicit content about the psychological impacts of a cancer diagnosis, and that it is normal to experience feelings of anxiety, grief, depression. You know all of the things that are not necessarily making you mentally ill, but are part of your journey, of having been diagnosed with an incurable cancer”. (Focus group 4) “I think if we were going to roll this out to patients ongoing, you want to try and make it a little bit more relevant to them. So her examples were beautiful, and we could relate to them”. (Myeloma Australia nurse)
	Simplification and rationalisation	“In the time that we had available, the amount of content and I think that maybe sort of trying to simplify it a little bit more, so that it gives a wider range of people, the ability to feel that they’re not left behind, like at times I felt like I was a bit left behind, and I kind of understand, you know, that we’re all going through the bumps and ups and bumps and ups and bumps. But it was really in a sense, I think somehow, maybe a kind of a less is more approach rather than trying to get it through as much as you did in that sort of 5 weeks”. (*Focus group 4*)
	Strategies for illness-related symptoms	“The only thing that I would say is that if it is going to be aimed at myeloma patients that you have to take into account, there will be times when people may drop out of sessions because of fatigue, or you’ve got such debilitating physical pain like bone pain, upset tummy, vomiting everything else that can, you know, accompany your drugs and that? But you have to allow there’ll be that flexibility. And I think that’s when it’s important that you’ve got those recorded sessions. So you can do that playback. I mean, even now I have completed this pilot program. I wouldn’t mind being able to tap back into those recorded sessions.” (*Focus Group 2*)
**Navigating Implementation: delivery, facilitation, and clinical integration** This theme explores the key implementation considerations for supportive care, focusing on effective delivery methods, the role of facilitation, program risks, and the integration of clinical practices	Clinical integration	“If there were nurses or specialists, or even volunteers that understood what this program was and then it was integrated into or offered and spoken about with knowledge of what the what the program provides at the start or during you know, so you had an understanding of what there is….. The nurses are all oncology nurses, they know basic stuff and would it be nice if they knew a little bit more about a plan like this”. (*Focus group 1*)
	Program timing and cohorts	“Information overload, coming to terms with your diagnosis all take time. I feel it can definitely help people with myeloma live well, as it can help put things into some perspective. Maybe if this was kept for those diagnosed 6 months or so might be a good time to introduce this. For me personally, this was the point I struggled with grief and loss”. (*Survey feedback*)
	Optimising program delivery	“And maybe some of this information could be uploaded into the database for Myeloma Australia so people can access themselves, you know a download that people can watch themselves again…. But I guess for me, I think it just essentially provides them a go to place to find the tools, to manage anxiety as best as they can”. (*Focus group 5*)
	Program risks for consumers and facilitator	“It’s a difficult one, my only one, and it’s not blaming anyone or anything, or whether there could be anything done about it. But when I was quite depressed, another person, not one of our fantastic group. Another person said she just decided not to feel bad anymore and I had to go off for a coffee. I was nearly in tears. I thought, I’m not making a decision to feel like this, you know, and I came back as she was still telling everyone about how to decide to feel better. But that’s not something that’s part of this. I think it’s when you’re with a big group that’s going to happen”. (*Focus group 1*) “It was quite a vulnerable space, and some of the participants were very open about their experience, and quite often it was also very emotional where people were tearing up when sharing, and then you could very much see also that the other participants wanted to obviously engage and share messages of hope and so navigating that space, I think, really required those skills and that and that knowledge”. (*Facilitator*)

#### Theme 1: Fostering peer connection and psychological strength

The *BWP* was reported to be a valuable and meaningful program that offered practical tools for mindfulness, reflection, and mental wellbeing, with consumers often describing notable emotional shifts such as moving from distress or anger to greater acceptance and gratitude. Many commented on how peer support helped reduce isolation and improved their skill development by practising and learning the skills and strategies alongside others in similar circumstances to themselves.

….It gave us a chance to meet other people who are living with this condition, and even though we’re all at very different places, someone might suggest something that you haven’t thought of, and vice versa, that can just help your days get better or be a bit better. And also it’s just the practical stuff. I just love the practice. (*Focus Group 1*).

These findings indicate that peer-based interactions and reflective components are key mechanisms for future adaptations.

#### Theme 2: Myeloma contextualisation: accommodations and additional myeloma content

Consumers emphasised the importance of myeloma-specific information and resources to better understand the disease and manage external stressors. Comments were also made about the need for simplified content, clearer pacing, and more consistent communication about the program’s purpose to reinforce the expected outcomes of the program.

…A couple of sessions were complicated and required your undivided attention. You must bear in mind that chemotherapy fog effecting concentration is a real thing. Also when you have days of physical pain, such as bone pain or severe fatigue attending the sessions would be very difficult. I am fortunate I am in remission so I could give my undivided attention. (*Survey feedback*).

Together, these findings indicate that clearer disease-specific psychoeducation, tailored myeloma-specific resources and refinements to session structure could improve the program purpose and acceptability.

#### Theme 3: Navigating Implementation: delivery, facilitation, and clinical integration

Consumers valued integrating mental health and wellbeing into routine care, emphasising promotion by nurses, collaboration with rehabilitation services, and alignment with bodies like Myeloma Australia.

…Don’t don’t try and do it separately. Try and bring it together in one space to me, and then you pick and choose. Each of us picks and chooses from the suite of things that’s on offering within that space. To me that makes sense. (*Focus Group 5*).

Both consumers and nurses acknowledged risks, noting potential distress for consumers and emotional burden for facilitators, highlighting the need for robust trainer support and clear strategies to manage group dynamics and sensitive content. These implementation findings highlight the need for streamlined and integrated mental health programs into existing reputable health services already to reduce the information overload and uptake readiness. In addition, structured facilitator support and clear guidance for managing group dynamics and distress should be considered for sustainable and safe delivery.

### Pre- and post-outcome measures

**Fig. 5:**
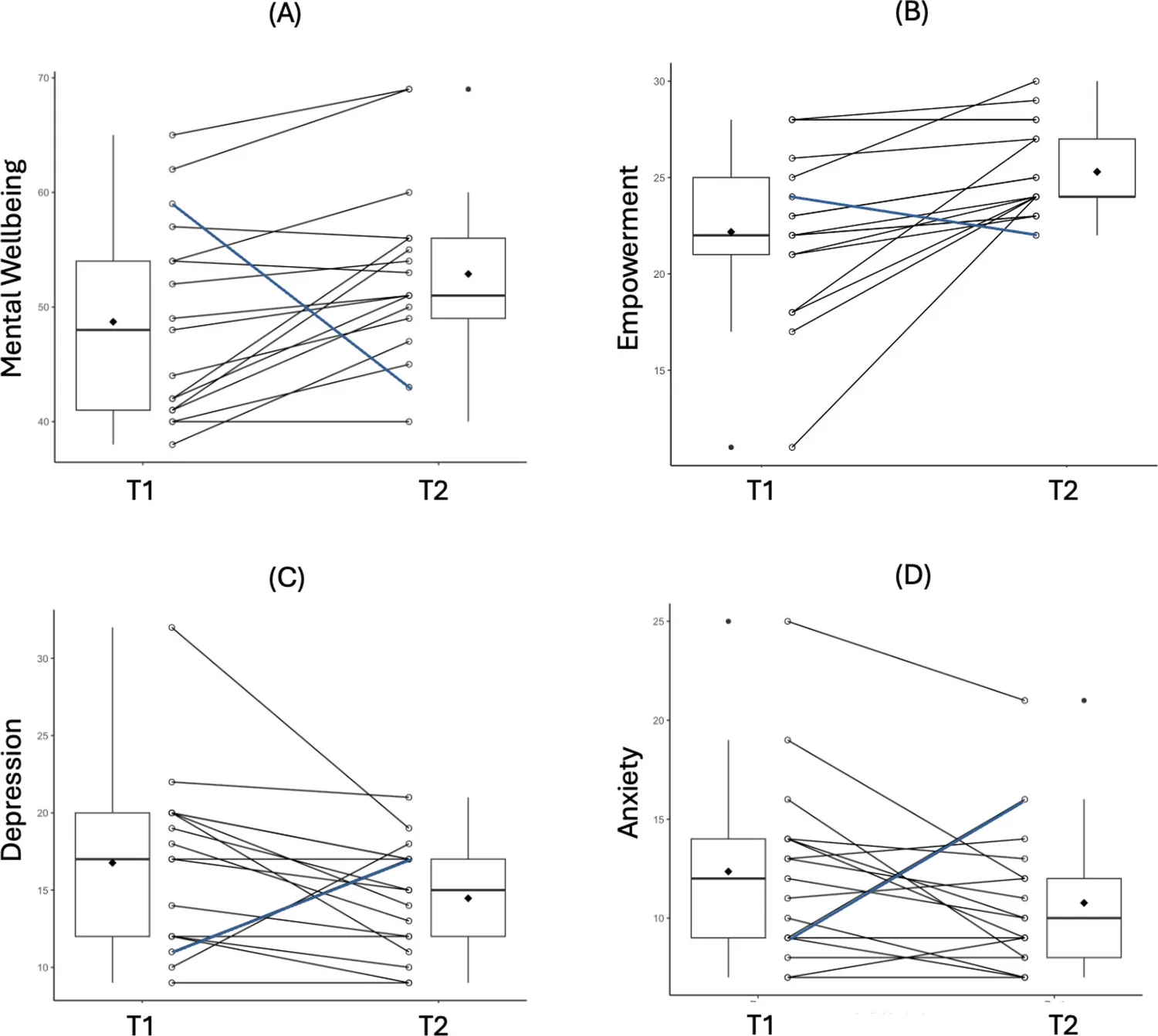
Pre- and post-outcome scores for complete-case outcome sample (*n* = 17). Box plots show the T1 (pre-) and T2 (post) distribution of scores; lines show individual consumer trajectories. **A** = Mental Wellbeing (WEMWBS), **B** = Empowerment, **C** = depressive symptoms (PHQ-9), **D** = anxiety (GAD-7). The blue trajectory indicates the consumer who experienced the intercurrent comorbid condition during the program (see S3)

Analysis between pre- and post-measures was performed on the entire sample who completed the pre-post-intervention outcome measures (*n* = 17; Fig. 5) A repeat measures MANOVA revealed a significant multivariate effect of time on the combined outcome variables, Wilks’ Λ = 0.481, *F*(4, 13) = 3.50, *p* = 0.038, partial *η*^2^ = 0.52. Follow-up univariate ANOVAs indicated significant improvements in both wellbeing (mean increase from 49.42 to 53.21) and empowerment (mean increase from 22.58 to 24.95) with large effects, with non-significant results for anxiety and depression (Table 3). Although changes in depression and anxiety did not reach statistical significance, effect sizes were in the medium to large range (*η*_p_^2^ = 0.153 and 0.120 respectively), suggesting the intervention may warrant further investigation in a larger adequately powered trial. The results were sensitive to the inclusion of one participant with an intercurrent comorbid condition. When exploratory analyses were repeated with the one individual removed, all outcomes demonstrated significant and large effect size differences at the univariate level (see Supplementary Table S3).

**Table 3:** Descriptive statistics for all outcomes pre- and post-intervention and univariate ANOVA tests (*n* = 17)

	Pre-intervention	Post-intervention	Univariate ANOVA		
	M (SD)	M (SD)	*F*	*p*	*η*_p_^2^
Wellbeing	49.42 (8.75)	53.21 (7.51)	5.78	0.027	0.243
Empowerment	22.58 (4.57)	24.95 (2.57)	5.88	0.026	0.246
Depression	16.84 (5.60)	14.74 (3.56)	3.24	0.089	0.153
Anxiety	12.05 (4.47)	10.79 (3.51)	2.46	0.134	0.120

*M* mean, *SD* standard deviation, *F* F-statistic, *p* probability value, *np*^*2*^ partial eta squared

## Discussion

Consumer-informed research plays a critical role in ensuring research leads to the development of effective, relevant, and acceptable interventions that address the needs and priorities of the people they are intended to benefit. Despite this, it is often overlooked or poorly executed [35]. In this study, we assessed the feasibility and acceptability of the evidence-based mental health intervention—the *BWP*—for consumers with MM and SMM and used consumer feedback to develop a series of recommendations to inform the future development of a tailored, myeloma-specific mental health and wellbeing program. Twenty consumers with MM or SMM and 4 Myeloma Australia nurses were involved across multiple stages from program delivery through to evaluation. Our study is consistent with recommendations from a recent review of consumer and research experiences in health research, particularly in relation to early involvement and engagement with consumers across multiple phases for inclusive involvement [36]. As such, this study acted as a pilot evaluation of the intervention through direct consumer participation prior to broader implementation. Although this approach was resource-intensive, it generated valuable insights that informed the refinement and future development of the intervention.

Although participant recruitment, satisfaction, and retention were encouraging, attendance rates indicate opportunities to improve engagement and accessibility prior to evaluation in a larger trial. A noticeable drop-off between initially expressing an interest in the program and commencing the program was observed with rural and regional consumers representing over half of the consumers who withdrew. This highlights a need to address barriers to uptake, including clearer upfront communication regarding program content, structure, and time commitment—an important consideration for future implementation as mental health service access for these populations may be more difficult [37]. Notably, consumers who attended at least three out of five sessions felt engaged throughout the study (75%) and all but one consumer participated in a focus group. This is an encouraging finding as the rate of engagement is consistent with the results from a breast cancer study using the same *BWP* program [12]. This contrasts with lower retention observed in some other psychosocial interventions among people with a hematologic cancers [38], where physical health demands were prioritised over mental wellbeing. A possible contributing factor to the high attendance in this study was the group-based, facilitator-led approach, which fostered peer connection and a sense of accountability. The variation in consumer responses highlights the importance of flexibility in program design. Individual factors, including age (e.g. younger consumers outside the typical myeloma demographic), comorbidities, cognitive functioning, treatment side effects, and prior mental health history, may influence engagement and the perceived benefit of the program. These findings reinforce the need for clearer program objectives, as well as adaptive, person-centred interventions [39, 40]. These factors point to key variables to explore in future randomised controlled trials.

Whilst this study was not designed or powered to test clinical effectiveness, self-reported outcomes demonstrated improvements in mental wellbeing and reductions in symptoms of anxiety and depression within participants. These results are important, as they provide valuable information to inform and adequately power a randomised controlled trial of the adapted *BWP*. Whilst these preliminary trends are encouraging, the results were impacted by a single participant who experienced a substantial health decline during the intervention period, resulting in the deterioration of mental health outcomes. Such events are not uncommon in the MM population and therefore represent an important consideration for future trial design. Notably, despite worsening mental health outcomes the individual continued to participate in the program and reported finding value in their participation during post-program follow up. This finding highlights the complexity of evaluating psychosocial interventions in populations who experience fluctuations in their physical health status.

Furthermore, the study demonstrated moderate to large within-person effect sizes across key psychological domains. Given the one-arm pre-post design, these findings provide preliminary signals for future controlled testing. The observed changes are consistent with findings from Zhang et al. [41], who reported similar effects in a myeloma-specific intervention incorporating evidence-based techniques such as behavioural activation, problem-solving, stress management, and strengthening social support. The alignment between these findings suggests that tailoring of interventions within the *BWP*, and social connection may enhance both engagement and clinical benefit.

From the qualitative feedback, consumers consistently described the program as beneficial, particularly valuing tailoring of mental health strategies, the opportunity to connect with others facing similar challenges and prioritise mental wellbeing within a supportive environment. Peer connection helped reduce feelings of isolation, echoing findings by Eilert et al. [13], who emphasised the therapeutic role of group identity in myeloma care. At the same time, many also reported acquiring practical skills to manage current and future challenges. This dual emphasis on immediate relief and long-term coping reflects best-practice principles in psycho-oncology interventions [42]. Although an adequate balance needs to be struck to ensure the program does not become a peer connection program, as some consumers expressed a preference for minimising peer discussions, noting a preference to focus on mental health strategies rather than hear detailed personal stories, which at times caused them distress. Consumers described increased self-efficacy, resilience, and supportive coping and building resilience, which aligns with a growing recognition that comprehensive cancer care must support both recovery from distress and the development of long-term psychological resources [43, 44]. Our future study will include an in-depth assessment of markers of positive function, beyond empowerment and mental wellbeing.

### Study strengths

A key strength of this study was the involvement of consumers throughout the entire research process, beginning with pre-implementation patient forums that raised the gap in mental health research and initiatives that directly informed this study design and ensured alignment with real-world needs. Consumers were actively involved in providing feedback throughout and post the program. The involvement of four Myeloma Australia nurses, and twenty consumers with MM and SMM including those from rural and regional locations, spanning a range of ages and time since diagnosis, throughout the study represents a robust sample for qualitative health research, especially within a rare cancer population. Although there is no universally defined sample size for co-design, existing literature supports the use of smaller, well-engaged groups to generate rich and meaningful insights without compromising feasibility [45]. Importantly, including both MM and SMM consumers is a novel aspect of this study, recognising the continuum of disease experience and the shared psychosocial challenges faced by these groups [7]. This inclusive approach will ensure that the mental health program developed is responsive to the diverse needs within the myeloma community.

The mixed methods design further strengthened the study by capturing both quantitative trends and rich qualitative narratives, which are essential for understanding the nuanced psychosocial needs of consumers and program preferences [46]. Additionally, including specialist nurses from Myeloma Australia provided valuable insight and practical guidance for tailoring the program to the myeloma community and as a future delivery partner, their involvement will help ensure the findings from this study will inform the development of an implementable program. The program achieved high levels of participation, with strong attendance and completion rates, as well as satisfaction rates among enrolled consumers. This sustained engagement reflects both the intervention’s relevance to the target population and the effectiveness of the consumer-informed process in developing a program that resonates with its intended audience.

### Study limitations

This study is subject to several limitations that must be considered alongside the positive signals of acceptability, feasibility and efficacy. First, the one-arm pre-post design, small complete-case sample, and absence of randomisation and a control group preclude causal inference regarding intervention effectiveness. Observed pre-post changes may reflect regression to the mean, natural fluctuation in symptoms, expectancy effects, or repeated exposure to the measures rather than the program itself. As this pilot study was designed to evaluate feasibility and acceptability rather than effectiveness, the outcome data should be read as preliminary signals warranting further testing rather than as evidence of effectiveness. Second, self-selection bias is likely; all consumers were all self-enrolled, identified as being Caucasian/European, and expressed interest in research. This homogeneity limits generalisability to more diverse populations, including those from different ethnic, socioeconomic, or educational backgrounds [47]. Partnering with experts in engaging minority populations will be important for ensuring broader uptake in subsequent work. Third, mental health outcomes in this population are shaped by factors well beyond the intervention. Some consumers disclosed that fluctuations in their mental health were influenced by physical health conditions and life stressors unrelated to the program. Self-reported measures in cancer populations warrant particular caution given the influence of medication side effects, disease progression and external stressors [48]. Fourth, the absence of long-term follow up means it is not possible to determine whether the observed changes were sustained beyond the immediate post-program period. Fifth, the dropout between expression of interest and program commencement may reflect uncertainty about what participation entailed, as reported by consumers in post-program feedback. Future studies should communicate the study's purpose and participation expectations more clearly at the point of recruitment [49]. Together, these limitations mean the present findings are best understood as establishing feasibility and identifying necessary adaptations, with a randomised controlled trial and longer follow-up required to establish efficacy.

### Summary

People with myeloma experience profound psychosocial distress, reflecting the high risk of progression, burdensome treatment side effects and, ultimately, the incurable nature of this disease. Here, we demonstrate encouraging feasibility and acceptability of the *BWP* and provide valuable insights into tailoring mental health interventions for MM and SMM populations, highlighting consumer-informed research in feasibility studies adds meaningful value to both psycho-oncology and implementation science. Actively involving consumers throughout the co-design process strengthened the relevance and acceptability of the intervention and established a foundation for future implementation at scale. Leveraging online delivery increased accessibility and uptake in diverse settings, while evaluating program implementation outcomes may enhance the likelihood of sustainable long-term improvements in mental wellbeing. Future research will utilise these insights to now co-design a tailored mental health program for people living with MM and SMM in a larger scale. Drawing on learnings from—among others—implementation science, these early findings affirm the value and rigour of deeply engaged, consumer-informed program development.

## Supplementary Information

Below is the link to the electronic supplementary material.

Supplementary file1 (PDF 150 KB)

Supplementary file2 (DOCX 20.1 KB)

## Data Availability

No datasets were generated or analysed during the current study.
