# Supplementary material for: Adapting a mental health program for people with multiple myeloma and smouldering myeloma: a consumer-informed feasibility study
Source: Support Care Cancer. 2026 Sep 8;34(10):937. doi: 10.1007/s00520-026-11163-2 (PMC13550037; doi:10.1007/s00520-026-11163-2)
Supplement: Supplementary file 1 [file 520_2026_11163_MOESM1_ESM.pdf]

Supplementary Tables

Table S1. Post quantitative feedback from consumers on the program aims, relevance of content, and overall satisfaction (n = 17).

	Strongly disagree n (%)	Somewhat disagree n (%)	Neither agree nor disagree n (%)	Somewhat agree n (%)	Strongly agree n (%)
I clearly understood the aims of the program	1 (5.9)	0	0	4 (23.5)	12 (70.6)
I felt engaged in the program	1 (5.9)	0	1 (5.9)	6 (35.3)	9 (52.9)
I learnt new information about my mental health and wellbeing	1 (5.9)	1 (5.9)	1 (5.9)	7 (41.2)	7 (41.2)
I learnt practical skills that help me to live with the challenges of myeloma	0	2 (11.8)	2 (11.8)	6 (35.3)	7 (41.2)
Please rate how much you liked the individual activities:					
In-session activities	0	0	4 (23.5)	12 (70.6)	1 (5.9)
Group discussions (n=1 missing)	0	0	3 (17.6)	7 (41.2)	6 (35.3)
Lecture format (i.e., learning the theory)	0	0	3 (17.6)	12 (70.6)	2 (11.8)
Breakout rooms	0	0	2 (11.8)	6 (35.3)	9 (52.9)
Using the workbook	0	0	4 (23.5)	7 (41.2)	6 (35.3)
Please rate how much you agree with the following statements about your facilitator:					

Knowledge of the materials	0	0	0	5 (29.4)	12 (70.6)
Preparedness	0	0	2 (11.8)	3 (17.6)	12 (70.6)
Time used effectively	0	1 (5.9)	3 (17.6)	5 (29.4)	8 (47.1)
Understanding/sensitivity of myeloma experiences	1 (5.9)	1 (5.9)	2 (11.8)	7 (41.2)	6 (35.3)
Effectively addressed complex and challenging questions	0	0	2 (11.8)	6 (35.3)	9 (52.9)
Encouraged participation and interaction	0	0	0	6 (35.3)	11 (64.7)

Table S2. Post-program quantitative feedback from consumers on the usefulness of strategies (n = 17).

	Not at all useful n (%)	Not very useful n (%)	Neither n (%)	Somewhat useful n (%)	Very useful n (%)
Learning practical skills for my day-to-day wellbeing	0	0	1 (5.9)	5 (29.4)	11 (64.7)
Learning practical skills to deal with more difficult times in life	0	0	1 (5.9)	7 (41.2)	9 (52.9)
Learning the different theories and building knowledge about mental health and wellbeing	0	0	1 (5.9)	10 (58.8)	6 (35.3)
Using the Be Well Tracker to understand about my mental health and wellbeing	0	0	3 (17.6)	8 (47.1)	6 (35.3)
Sharing my experiences with others in breakout rooms	0	0	0	6 (35.3)	11 (64.7)

Practicing the skills I learnt in between sessions	0	0	3 (17.6)	8 (47.1)	6 (35.3)
Learning about how to develop habits	0	1 (5.9)	0	10 (58.8)	6 (35.3)
Self-reflection on what works for me and what I can improve on	0	1 (5.9)	0	8 (47.1)	8 (47.1)
Establishing relationships with other people living with myeloma	0	1 (5.9)	1 (5.9)	5 (29.4)	10 (58.8)

Table S3. Pre- and post intervention outcome measures without the individual outlier (n=16)

	**Pre-intervention**	**Post-intervention**	**Univariate ANOVA**		
	M (SD)	M (SD)	F	p	η_p^2
Wellbeing	48.06 (8.44)	53.50 (7.69)	21.77	0.001	0.588
Empowerment	22.06 (4.58)	25.50 (2.31)	14.18	0.002	0.486
Depression	17.13 (5.70)	14.31 (3.66)	6.24	0.025	0.294
Anxiety	12.56 (4.70)	10.44 (3.56)	8.61	0.010	0.365

Note: A repeat measures MANOVA revealed a significant multivariate effect of time on the combined outcome variables, Wilks' Λ = 0.364, F4, 12) = 5.251, p = .011, partial η^2 = 0.636. Follow-up univariate ANOVAs indicated significant improvements in all four outcomes with large effects.

Table S4. Illustrative quotes for sub-themes and codes

**Theme: Fostering peer connection and psychological strength**		
**Sub-theme: Skills and strategies to support coping and building resilience**.	**Code**	**Illustrative quote**
The development of essential skills and strategies aimed at enhancing coping mechanisms and building resilience was noted among consumers. Individuals reported feeling better prepared to face the ongoing challenges associated with myeloma and relapse. Consumers particularly valued the incorporation of mindfulness practices and practical tools to reduce stress and anxiety. These resources collectively improved their confidence in managing their condition and navigating the complexities of their journey with greater emotional strength and resilience.	Resources to build confidence	"The other thing was to. I saw it as a way of improving my resilience, and someone at the very last session talked about being ready. And I like that idea that I know I've got time now in the good times where I can try and get my head around. So when I do go through some bad times, I've got something to to lean on, and that's actually a bit soothing for me. In the fear that I've got it feels like you with the tools that we've got in the back of this booklet, and I reckon having the booklet itself is an amazing resource, but it does help give your mind some respite from the whole thing, and having a break from it, so utilizing some of those tools." (Focus Group 5)
	Benefits of mindfulness	"I think the skills that I practiced was to be as mindful as I could in the simple things that I was doing. So I think that being able to focus on just being in the moment was probably one of the most helpful skills that that I developed." (Focus Group 2)

	Feeling prepared for the next big challenge	“I know that I will keep this in my mind when I hit my next big problem, and that's what's so useful for myeloma. Because, as people have said before, as well you know, you're going to get to a big problem. You just don't know when. And that is very stressful. So you have to work towards trying to live your life in the moment without having to think about those things coming. So all this sort of stuff is very, very important for that.” Focus Group 2)
**Sub-theme: Behavioural activation and motivation.**	**Code**	**Illustrative quote**
The program was seen as providing direction on how to apply previously learned wellbeing skills, making these strategies feel more accessible and actionable. Incorporating daily practices was especially valued, as it helped embed mental health routines into everyday life, creating a sense of structure and ongoing commitment to self-care.	Self-compassion	“So I've gone and done a Pilates class, and I'm starting to just be kinder to myself in terms of that negativity when I, when I haven't done anything that. So that goal setting particularly things like try and back it on to another habit that you've got. I probably pinpointed in myself fear that I didn't realize that I was carrying.” (Focus Group 5)
	Gave a sense a direction of to improve wellbeing	“I have found it incredibly helpful. it's also helped me with a lot of my reflections on the things that are most important. For you know, just really, in a sense, planning my year ahead as best I can.” (Focus Group 4).
	Daily practices	“Adding small things to activities I already do to make it that I don’t have to think about them but they’re just party if life.” (Survey Feedback)
	Prioritising mental health	“I worked for over 30 years with people who needed support, I worked in aged care, disabilities and mental health. The last 5 years I was very lucky to have worked as a registered nurse in mental health. The Be Well plan

		strongly indicated to me that now is the time to start looking after myself." (Survey feedback)
**Sub-theme: Learning through connection**	**Code**	**Illustrative quote**
Consumers highlighted that learning through connection and peer support helped reduce isolation, fostered collaboration, and enhanced skill development by practicing and learning the wellbeing skills and strategies alongside others in similar circumstances to themselves.	Peer support	"It was good just to be part of a group just coming together for every week. I think that in itself, even despite any of the content kind of felt like nice to get together and be a part of something." (Focus Group 1)
	Alleviates isolation	"I found it helpful that others are feeling the same way, and are having mental health issues over the same thing over this horrible disease. Sometimes you might think of yourself as being a bit weak. I'm letting this get on top of me. It's now affecting my day-to-day activities. It's sad, but it's nice to know that you're not alone out there that others are feeling exactly the same way." (Focus Group 2)
	Learning skills with others	"I think from that you meet other people that are going through a similar journey or a journey with the same disease and you will learn some really good ideas and and tactics for building the strength." (Focus Group 1)
**Theme: Myeloma contextualisation: accommodations and additional myeloma content**		
**Sub-theme: Myeloma specific information and resources**	**Code**	**Illustrative quote**
Consumers expressed a desire for information specifically tailored to myeloma to help them better understand the disease. This included resources to	Improve knowledge about the disease	"Is it possible to have some pre-sessions where there's a bit of information, you know, or something prior to this, although I guess most of us like someone that's already got the fully fledged disease. You know, we kind of

manage external financial stressors, support in communicating with family members, and a deeper understanding of why mental health support is a necessary part of living with myeloma - not simply a reflection of personal weakness.		already on that journey. So we've had to work out our own way around it. So would it be helpful you know, for us to share some of that stuff in some way, shape or form don't know." (Focus group 5)
	Resources for external stressors	"Also like resources, for you know how to approach things with your family, because, you know, it impacts everyone around us as well. I've had a whole list of concerns about, you know finances and things like, if I'm not around, you know the main income, the one who does things around the house kind of guy, you know, all, all that kind of stuff." (Focus Group 6)
	Context about why mental health support is relevant to myeloma	"I feel that the wellbeing focus on mental health only, overlooks what causes us to suffer with our mental health. That is, pain and the various intrusive procedures we go through. A session on how we all have dealt with pain… living with chronic pain and or pain that is associated with procedures." (Survey feedback)
**Sub-theme: Simplification and rationalisation**	**Code**	**Illustrative quote**
Consumers highlighted the need for simplified content and increased repetition across sessions. Many found the two-hour session length difficult to sustain, yet also felt the sessions were rushed, leaving insufficient time to fully engage with the workbook activities and content. There was a clear call for more structured pacing, along with better upfront communication about the program's	Clarity on the overall purpose and intended outcomes	"And it was only later on, probably about the 3rd or 4th session that I realised where there was all this was going. You know that we're trying to find out outcomes we want, you know, like this is on pages 48, 49, and 50. I think that's a critical part of the whole book. In some ways we were trying to find out where we're weak, what sort of things we need to work on, and then what activities relate to it, and then go to the activity finder to see which ones are relevant. And so that logic was there, probably, and it was

overall purpose, specifically, its aim to support the development of an individual mental wellbeing plan and clearer guidance around homework tasks.		probably explained several times. But I didn't pick it up until about the 3rd or 4th session that that's where we were going.” (*Focus Group 2*)
	Simplification of concepts with more repetition	“I actually thought some of the theory was very intense. And the reason why I say that is that while I could concentrate on it, [the facilitator] you know she'd give wonderful presentations, but it was something that I'd have to go back and digest again before I could do the homework activities.” (*Focus Group 2*)
	Rationalisation of the volume of information	“I think maybe a couple of the activities might have needed a little bit longer to felt like, particularly the apps, you know, trying to get onto the app and complete the tracker and all that sort of stuff. Whether maybe we've done that before the session, or I think that that was, I felt that was a bit rushed for me to try and read it properly and get my head around it, and all of that.” (*Focus Group 3*)
**Sub-theme: Strategies for illness related symptoms**	**Code**	**Illustrative quote**
Living with symptoms such as pain, fatigue, and gastrointestinal issues were described as a significant contributor to consumer’s mental health struggles. Suggestions included using the program to support coping with these stressors and adapting session delivery to better accommodate individuals actively experiencing these physical challenges.	Living with pain and fatigue	“The only thing that I would say is that if it is going to be aimed at myeloma patients that you have to take into account, there will be times when people may drop out of sessions because of fatigue, or you've got such debilitating physical pain like bone pain, upset tummy, vomiting everything else that can, you know, accompany your drugs and that? But you have to allow there'll be that flexibility. And I think that's when it's important that you've got those recorded sessions. So you can do that playback. I mean, even

		now I have completed this pilot program. I wouldn't mind being able to tap back into those recorded sessions.” (*Focus Group 2*)
	Grief of physical decline	“I certainly feel this personally is that I have suddenly aged in the sense that you know, 5 years ago. I was extremely fit and extremely capable. The world was my oyster, you know. We were traveling overseas, doing all sorts of things, and my physical capability has taken, if you like, a sort of a 10 to 15 year aging hit, you know. So I'm like you know, I can imagine like, I'm like a sort of a 75 year old in terms of physical capability, and that is a mental health issue.” (*Individual Interview*)
**Theme: Navigating Implementation: delivery, facilitation, and clinical integration**		
**Sub-theme: Clinical integration**	**Code**	**Illustrative quote**
Integrating mental health and wellbeing into clinical practice and services was seen by consumers as valuable and they emphasised the importance of promotion by nurses and other health professionals, potential collaboration with physical rehabilitation services, and alignment with professional bodies such as Myeloma Australia to ensure cohesive and comprehensive care delivery.	Promotion by health professionals	“I think it's the nursing staff that need to be made aware of this to be able to get the information out to patients. So if we could have the input from them as well as input from myeloma, Australia nurses to get the information out there as well. Not everyone who's being diagnosed has a touch point with the myeloma nurses. It's the hospital nurses. Hospital infusion suites that in the initial stages, get our information from so having them be aware that to guide patients towards this, I think, would be fantastic.” (*Focus Group 4*)
	Integration with physical rehabilitation	“After an extended period in hospital with radiotherapy and stem cell transplants, many multiple myeloma patients are assigned to a physical rehab program. This must cost a huge amount, but illustrates a willingness

		to ensure that they can literally get back on their feet. Just as important is mental health rehab, and parallel investment in face to face small group and 1 on 1 sessions should be paired with physical rehab." (*Individual Interview*)
	Integrate with professional bodies	"I actually didn't probably didn't read between the lines that always about you know mental health, mental wellbeing and I wonder if somehow or other it is part of the package. So through Myeloma Australia and or the Cancer Council who have that myeloma kind of stream. But when you contact, for example, Myeloma Australia, here's the resources. Here's the information booklets, there's some really good booklets online with Myeloma Australia and good podcasts." (*Focus Group 5*)
**Sub-theme: Program timing and mixed cohorts**	**Code**	**Illustrative quote**
Consumers shared mixed views on including individuals with SMM and myeloma in the same program, with some expressing empathy while others questioned the fit. There was general agreement that the program is most beneficial either at diagnosis whether SMM, or myeloma, or after initial treatment when individuals are adjusting and anticipating potential relapse. Myeloma nurses raised that group dynamics influenced delivery, with some participants treating the sessions as informal	Considerations for combining myeloma and smouldering myeloma	"And that's where my empathy started because I was. I think I've got another friend who's smouldering, and I think I was very mindful of what information was going on. I felt for him and it, and it kind of stopped me for a little while. Because you kind of want to help him. But you can't." (*Focus Group 1*)
	Alignment of participation with key events	"It's like it's like a timing thing. You know what I mean. So it's like when all your treatment's done, and you know, getting zomat every month, or whatever it is probably, then, is a good time to do something like this, because it's like a flowing on from your treatment to, you know 'you don't

support groups, highlighting the importance of clear facilitation and boundaries.		know what to expect', 'you know I'm in remission but what is it is going to come back?'. You know, so that help mental health side of it, I think will help from that point of view for me." (*Focus Group 2*)
	Introduce at the time of diagnosis of myeloma or SMM /MGUS	"I think this would have limited use at initial diagnosis if going immediately onto treatment. Having to coordinate appointments and treatment is all time consuming that adding another 'thing' to have to do people wouldn't get the full benefit. And you then also throw chemo brain into the mix. Likewise, if you are years down the track, much of what was in the program you've already worked out. I believe a sweet time for this to be most beneficial is - soon after being given the diagnosis of MGUS or SMM if treatment is not going to be commenced and you are 'monitored." (*Survey Feedback*)
**Sub-theme: Optimising program delivery**	**Code**	**Illustrative quote**
Consumers highlighted the importance of optimising program delivery through flexible modality options, thoughtful group size, and effective use of online platforms. Skilled facilitation was also seen as critical to creating a supportive and engaging learning environment. Myeloma nurses raised practical considerations included reducing group size, shortening and spacing sessions over a longer period, and	Flexible modality options	"And maybe there could be some of this information could be uploaded into the database for Myeloma Australia. So people can access themselves, even you could. Do you know a download that people can watch themselves again? Just a general overview of what it's all about. And then they could then register for it if they felt it was useful for them." (*Focus Group 5*)
	Group size and online considerations	"Smaller group and no break out rooms so everyone gets to know everyone better and gets to tell their story better. I only had 2 others in my group and

encouraging reflection outside the group to maximise learning and engagement. The nurses also highlighted the value of offering follow-up services from Myeloma Australia to sustain benefits.		it was a bit awkward at times. Session one should be everyone having 5mins to tell their mm story." (*Survey feedback*)
	Facilitation skills	"I liked that it was done by a psychologist because it actually gave it more I don't know, there seemed to be a broader understanding. But whoever has it has that also that compassion and understanding of myeloma that resonated also the Myeloma Australia Nurse Coordinators, someone like that could probably also run similar things." (*Individual interview*)
**Sub-theme: Program risks for consumers and facilitators**	**Code**	**Illustrative quote**
Engaging in the program could evoke emotional distress for some, with concerns raised about the potential for mental health deterioration during or after participation, as well as the emotional burden placed on facilitators supporting individuals through challenging content. The facilitator recommended appropriate support for the trainer to ensure they could respond sensitively to the vulnerabilities and unique needs of participants, including guidance on navigating the dynamics of the group and responding to those expressing psychological distress.	Emotional distress for consumers from participating in the program	"I'm really only just speaking as one person who did, you know, through a period of feeling, more stress and anxiety through the program, found that I was kind of feeling a bit stressed because it wasn't working as well as I hoped it would, and even though it was made clear that you know it might not work in all situations, but but in the end, you know, that was really where I bumped along the bottom a bit." (*Focus Group 4*)
	Risk of consumers' mental deterioration during and post program	"I also had a major depressive episode soon after the cyclone which lasted for more than a few days. My wife thinks I was just exhausted, and the depression was a result. I have had depression in the past, but more than 15 years ago. I struggled through session 4 and had to walk away a few times. I think these experiences skewed my experience and tracker results." (*Survey Feedback*)

	Emotional burden for the facilitator	“I also think I certainly think that whoever is going to be the facilitator should be provided with clinical supervision afterwards, and on a very regular basis, because it was very emotional to be quite honest, definitely after the session, like it's, you know, they're sharing very sad stories. And you need to be able to work through them and you know whether they are maybe activating anything in yourself.” (*Facilitator Interview*)
