# Supplementary material for: Adapting a mental health program for people with multiple myeloma and smouldering myeloma: a consumer-informed feasibility study
Source: Support Care Cancer. 2026 Sep 8;34(10):937. doi: 10.1007/s00520-026-11163-2 (PMC13550037; doi:10.1007/s00520-026-11163-2)
Supplement: Supplementary file 2 [file 520_2026_11163_MOESM2_ESM.docx]

**Supplementary Methods**

*Focus Group Semi-Structured Interview Questions*

1. Impact of the Be Well Plan:
   a. We are interested to hear about your experiences of the Be Well Plan

- What were your experiences of the *BWP?*
- *Prompt: In what way, if any, did the BWP program change the way you approach the stressors or challenges you face with living with myeloma?*
  b. We are interested in understanding what aspects of the *BWP* were helpful to you and those which weren’t.
- What specific aspects of the *BWP* did you find helpful for managing the concerns you have living with myeloma? Please describe these.
- *Prompt: What skills did you find the most helpful?*
- *Prompt: how valuable were the skills compared with the social interaction?*
  c. Were there any specific aspects of the *BWP* that you found *unhelpful* for managing the concerns you have living with myeloma? Please describe these.

2. Utility of the BWP for people living with myeloma:
   a. We are interested in understanding your perspective in how the *BWP* could be useful for people living with myeloma.

- Do you feel the *BWP* can be helpful for people who are living with myeloma? How?
  b. We are also interested in better understanding how to adapt the *BWP* to be more valuable for people living with myeloma.
- Do you feel changes would need to be made to the *BWP* to support people living with myeloma? If so, what type of changes?
- *Prompt: What myeloma specific content would you expect?*

3. Implementation
   a. We are also interested in understanding why people with myeloma would be interested in a mental health and wellbeing program.
   b. What would entice people living with myeloma to register for the *BWP*?
   c. How can we prepare people for program?
   d. In an ideal world, how would the *BWP* program integrate with your current supportive care?

*Program evaluation and feedback survey*.

Open-ended questions included “What was the main thing you took away from the Be Well Plan?” and “How do you think this program could support people to live well with myeloma?”. Consumers also responded to Likert-scale items (rated from strongly disagree to strongly agree), such as “I clearly understood the aims of the program”, as well as usefulness ratings (ranging from not useful at all to very useful), for example “Learning practical skills for my day-to-day wellbeing”. These items assessed clarity of program aims, relevance of content, usefulness of strategies, and overall satisfaction with the program. Together, the open- and closed-ended questions provided complementary insights into consumers’ experiences and perceptions of the program.

Q1. What was the main thing you took away from the Be Well Plan?

Q2.Was there anything you liked in particular about the Be Well Plan?

Q3. Was there anything you did not like in the Be Well Plan?

Q4. Is there anything we could improve?

Q5. Do you have any comments about the facilitator? (e.g., knowledge and expertise, engagement and communication, preparation and organisation, overall effectiveness)

Q6. Which activity or activities did you practice most during the program? And do you think you will continue to use them?

Q7. How do you think this program could support people to live well with myeloma?

Q8. Do you have any other comments or feedback?
